# Modified Maxillary-Swing Approach for Resection of Primary Malignancies in the Pterygopalatine Fossa

**DOI:** 10.3389/fonc.2020.530381

**Published:** 2020-11-09

**Authors:** Li Xie, Wenxiao Huang, Junqi Wang, Yue Zhou, Jie Chen, Xue Chen

**Affiliations:** ^1^Department of Head and Neck Surgery, Hunan Cancer Hospital, Xiangya School of Medicine, Central South University, Changsha, China; ^2^Department of Radiation Oncology, Hunan Cancer Hospital, Xiangya School of Medicine, Central South University, Changsha, China

**Keywords:** malignant tumor, *en bloc*, modify, maxillary swing approach, pterygopalatine fossa

## Abstract

**Background:**

*En bloc* resection of malignancies in the pterygopalatine fossa (PPF) poses critical challenges. Using the modified maxillary-swing (MMS) approach, we achieved monobloc removal of primary malignancies in this region. This study provides a detailed account of the surgical techniques and indications used.

**Methods:**

We enrolled seven patients with primary malignancies in the PPF during a period from January 2012 to January 2019 in this retrospective study. After malignancies were confirmed by biopsy as well as evaluation with computed tomography (CT) and magnetic resonance imaging (MRI) scans, all of the patients underwent MMS surgery under general anesthesia to extirpate these tumors. We performed regular postoperative follow-up using CT and MRI scans.

**Results:**

*En bloc* resection was successfully performed in all cases. We observed negative margins in six cases and positive margins in one patient with adenoid cystic carcinoma, who received postoperative radiotherapy. The most common complication was facial numbness. During the follow-up period (range, 6–69 months), one patient suffered from recurrence, while the others did not.

**Conclusion:**

The advantages of the MMS include a wide surgical field, full exposure, and easy manipulation. We expect this approach to become an alternative to the monobloc resection of malignancies in the PPF that involve the infratemporal fossa, maxillary sinus, nasal cavity, orbit, or oral cavity.

## Introduction

The pterygopalatine fossa (PPF) is a region marked by complex anatomy. Malignancies originating in this area pose a therapeutic challenge to surgeons due to its proximity to vital structures and limited exposure, making manipulation dangerous. Many surgical approaches have been designed to maximize exposure and minimize damage to the neurovasculature. Wei et al. first described the maxillary-swing approach for persistent or recurrent nasopharyngeal carcinoma in 1991 (1). Sumi et al. (2) and Otremba et al. (3) later documented that this procedure also provided wide exposure of the PPF and facilitated proper clearance of lesions. However, they did not take into account the following two facts: (1) the posterior osteotomy behind the maxillary tubercle inevitably involves a close margin, sometimes even resulting in tumor rupture and spillage (2, 4) and (2) the surrounding canals and foramina (e.g., infraorbital fissure, sphenopalatine foramen, and greater palatine foramen and canal), which probably serve as sanctuary sites for tumor cells, are left undisturbed by conventional bony cuts (5). To overcome these problems, we introduced a modified maxillary-swing (MMS) approach for the monobloc resection of primary malignancies in the PPF.

## Method

The modified procedure and the retrospective chart review were approved by the Independent Ethics Committee of Hunan Cancer Hospital, Changsha, China.

## Patient Demographics

From January 2012 to January 2019, seven patients who suffered from biopsy-confirmed primary malignancies in the PPF without any cervical or distant metastasis underwent MMS. This group included three male and four female participants, with a mean age of 46.3 years (range, 13–67 years). We routinely included computed tomography (CT) and magnetic resonance imaging (MRI) scans as well as laboratory tests in our preoperative assessments. Our decision as to whether to implement the MMS or another surgical procedure depended on the patient’s written informed consent after surgeons had thoroughly explained the benefits and possible complications. Patients were excluded from this study for refusal or inability to tolerate curative surgery, the involvement of the retrostyloid space, or intracranial extension.

## Surgical Procedure

**FIGURE 1:**
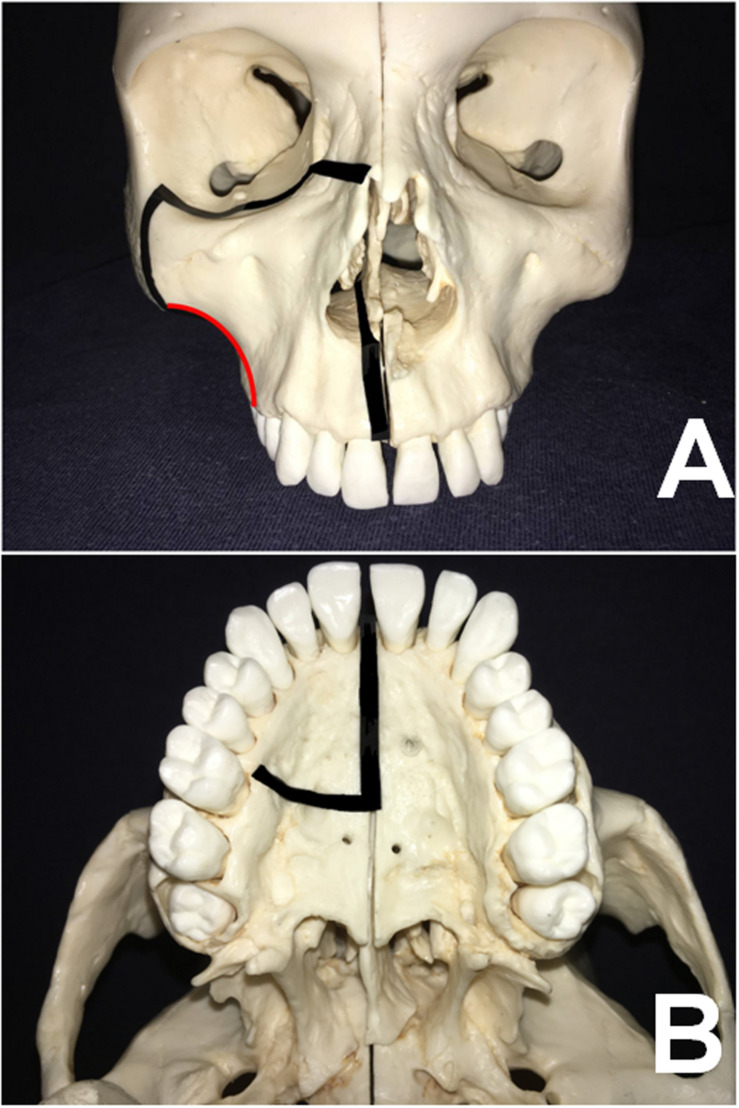
**(A,B)** Based on the skull model, the osteotomy lines of the MMS approach are illustrated in the axial and coronal views, respectively. (The red arc indicates the osteotomy lines on the facial ridge).

**FIGURE 2:**
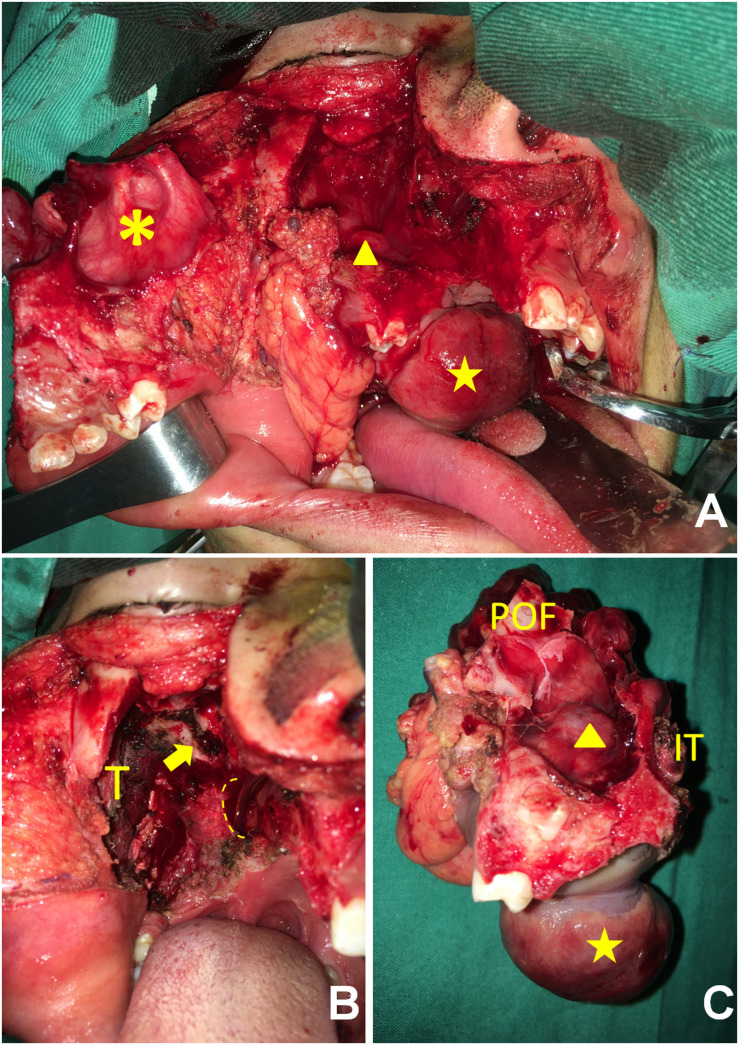
**(A)** The anterior maxilla (asterisk) was rotated laterally, providing wide exposure to the tumor protruding into the sinus (triangle) and the oral cavity (star). **(B)** After tumor extirpation, the root of the pterygoid process (arrow), the choanae (dotted line), and the musculature of the infratemporal fossa were exhibited. T, temporalis. **(C)** The anterior view shows that the tumor had invaded the maxillary sinus (triangle) and the oral cavity (star). IT, inferior turbinate; POF, posterior orbital floor.

Patients underwent general anesthesia with nasotracheal intubation via the contralateral nostril. The patient was placed in the supine position. Ipsilateral tarsorrhaphy was routinely employed while the contralateral eye was covered. At the very beginning, through a 5-cm transverse incision along the dermatoglyph in the upper neck, we clamped the ipsilateral external carotid artery to reduce blood loss during the subsequent procedure. The surgical procedure started with a Weber–Ferguson incision that extended along the nasal contour to the medial canthus with a midline split of the upper lip to the base of the columella and then deviated to the infra-orbital lateral extension on the side to be exposed. The skin incision was deepened through the soft tissues and musculature until it reached the periosteum. The anterior soft tissue of the cheek was elevated minimally to expose the following underlying bony structure: the surface of the zygoma, the inferior orbital rim, and the frontal and alveolar processes of the maxilla. We made a palatal incision at midline and turned it laterally to the gums between the second premolar and the first molar. We used Wei’s method, but with two technical modifications: (1) Coronal osteotomy was performed at the facial ridge and hard palate (HP), instead of at the hamulus of the pterygoid, to preserve the integrity of the posterior and posterolateral walls of the maxillary sinus (MS) and (2) parts of the maxilla, the orbital floor (OF) and infraorbital rim were swung simultaneously (Figure 1). At the zygomatic process, we inserted the oscillating saw through the MS in a horizontal position (along the transverse mucosal incision) to fracture the anterior maxilla and HP. Osteotomies were continued at the frontal process and the midline of the HP. We positioned a splitting chisel and drove it inward to the osteotomy line to separate the bony connection. The anterior maxilla could be retracted laterally with the facial skin, resulting in a broad view of the PPF (Figures 2A,B). We were then able to pay attention to the primary tumor. The medial pterygoid muscle was detached from the mandible, followed by transection of the lateral pterygoid muscle. We used the chisel to fracture the pterygoid process (PP). Finally, we removed the tumor in monobloc fashion together with the contiguous structures [the sinus posterior and posterolateral walls, the HP, the lateral wall of the nasal cavity (LWNC), and the PP and muscles]. As advocated by some experts (6, 7), we used a vascularized free flap to reconstruct such large defects (the lateral nasal wall, partial OF, and HP). The pedicle of the flap was tunneled through the subcutaneous soft tissue of the cheek to the neck for subsequent microvascular anastomosis. We then rotated the laterally swung maxilla back to its normal anatomic position and fixed it to the zygoma and the frontal and alveolar processes using miniplates and screws.

## Postoperative Care

To decrease flap failure, we routinely performed postoperative monitoring of the free flaps every 2–3 h during week 1 postsurgery. Patients’ noses were unilaterally packed with iodoform gauze for 5 days to keep the free flap in position. The use of postoperative antibiotics is recommended in the first 7 days.

## Follow-Up

We followed up with all patients every 3 months during the first 2 years, every 6 months during the next 3 years, and annually thereafter. Computed tomography, MRI, and positron-emission tomographic (PET) CT were used to detect any residual or recurrent disease if necessary.

## Results

**FIGURE 3:**
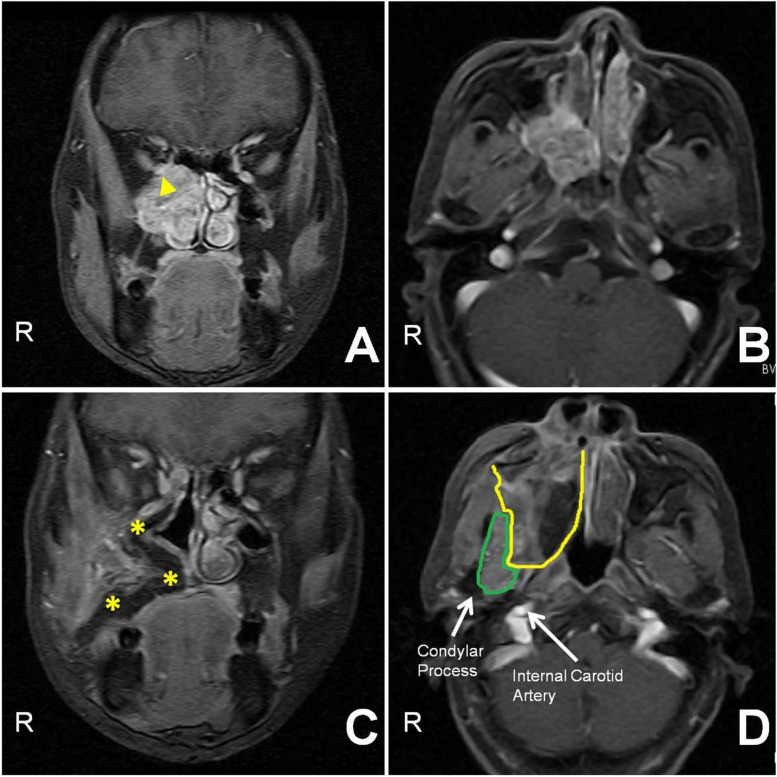
**(A,B)** Preoperative contrast-enhanced coronal and axial T1-weighted MR images show that a tumor (triangle) occupied the PPF, invading the orbit and nasocavity. **(C)** Thirteen-month postoperative contrast-enhanced coronal T1-weighted MR image demonstrates that the flap (asterisk) supported the orbital contents and covered the defects without recurrence. **(D)** The postoperative contrast-enhanced axial T1-weighted MR image shows that the maxillary-swing approach failed to resect the lateral part of the infratemporal fossa (referred to as the “blind spot,” bordered in green; the flap is encircled by the yellow line).

All of the patients’ demographic characteristics, tumor characteristics, pathological findings, and follow-up outcomes are summarized in Table 1. During surgery, we found that all of the neoplasms had irregular shapes, incomplete capsule or pseudocapsule, hard consistency, and good vascularization status. All of them had aggressively invaded neighboring bony structures such as the PP, the posterior and posterolateral walls of the maxillary antrum, the LWNC, and the HP (Figures 2C, 3A,B). Mean operation time was 8 h (range, 6.5–10 h); the mean length of hospital stay was 10 days (range, 7–20 days). The mean amount of intraoperative bleeding was 200 ml (range, 100–400 ml). Gross monobloc resection was achieved in all cases, while negative microscopic margins were obtained in six. Because of the large bone and soft-tissue defects, all patients underwent immediate free-flap transfer reconstruction following tumor resection. In the current study, the workhorse flap was an anterolateral thigh flap for six patients and an anteromedial thigh flap for the remaining patient. One patient (case 3) with adenoid cystic carcinoma who showed microscopically involved surgical margins of the maxillary and infraorbital nerves was recommended to undergo postoperative radiotherapy at a moderate dose (66 Gy) within 6 weeks after discharge.

**TABLE 1:** Summary of demographics, tumor characteristics, pathological findings, and follow-up outcomes of all patients who underwent surgery using the MMS approach.

Case no.	Pathology	Age (years), sex	Presentation	Size, location and extensions	Follow-up	
					Months	Outcomes
1	Mucoepidermoid carcinoma	50, M	None	5 cm × 3 cm × 3 cm, right PPF and ITF, PP, HP	69	No recurrence
2	Mucoepidermoid carcinoma	67, F	Intermittent headache	6 cm × 5 cm × 4 cm, left PPF and ITF, IOF, PP, HP	54	No recurrence
3	Adenoid cystic carcinoma	45, F	Facial numbness	3 cm × 3 cm × 3 cm cm, left PPF and ITF, PP, LWNC	41	No recurrence
4	Myofibrosarcoma	59, F	Mild headache	4 cm × 4 cm × 3 cm cm, right PPF and ITF, IOF, OF, PP, LWNC, MS	25	No recurrence
5	Fibrosarcoma	13, M	Palatal protrusion and numbness	7 cm × 5 cm × 5 cm, right PPF and ITF, PR, HP, MS, OC	24	Local recurrence after 1-year follow-up, resected again, then no recurrence
6	Carcinoma in pleomorphic adenoma	40, M	Mouth angle Numbness and headache	4.5 cm × 4 cm × 3 cm, right PPF and ITF, IOF, OF, HP	16	No recurrence
7	Carcinosarcoma	50, F	Palatal protrusion and stuffy nose	3 cm × 3 cm × 3 cm, left PPF and ITF, HP, LWNC	6	No recurrence

*HP, hard palate; ITF, infratemporal fossa; IOF, infraorbital fissure; LWNC, lateral wall of the nasal cavity; MS, maxillary sinus; OC, oral cavity; OF, orbital floor; PP, pterygoid process; PPF, pterygopalatine fossa.*

Regarding complications, all patients experienced expected postoperative facial numbness and epiphora. However, they exhibited varying degrees of relief within 6 months. Case 5 presented mild malocclusion postoperatively, but this deformity did not evolve into a functional disturbance during follow-up. Other morbidities, such as trismus, palatal fistula, facial paralysis, and diplopia, were absent in this cohort. The scars in the facial region are nearly invisible.

Patients widely complied with regular follow-ups. Postoperative imaging showed favorable outcomes for the reconstruction of soft-tissue defects and bone loss using the vascularized free flaps in the retromaxillary region (Figures 3C,D). The contents of the PPF and infratemporal fossa (ITF) were completely extirpated except for the lateral part of the ITF (Figure 3D). Of these seven patients, only one (case 5) showed recurrence in the ITF at the last follow-up visit and underwent surgical treatment at another tertiary hospital, while the other six are alive and disease-free.

## Discussion

The PPF is a relatively small and concealed area that communicates with intracranial and extracranial compartments via multiple bony canals and foramina through which different neoplasms can spread back and forth (5). The heterogeneity of the PPF’s tissues makes it a bed for a wide spectrum of benign and malignant lesions with variable prognoses (8), but its anatomical complexity poses a challenge to surgeons who hesitantly commit to removing such lesions.

Several routes to the PPF have been explored. They are divided into three types: lateral, inferior, and anterior. The lateral approach, mainly referred to as the ITF approach, was pioneered, elaborated upon, and implemented by Fisch (9). It provides sufficient exposure to the PTF, the ITF, and the great vessels in the retrostyloid space, but it cannot offer good visualization of any tumor exceeding the midline. Additionally, this procedure requires mastoidectomy and transposition of the facial nerve, leading to postoperative conductive-hearing loss and neurological deficits. The inferior method, the so-called transmandibular approach, achieves only a wide view of the anterolateral compartment of the ITF and pterygoid hamulus. In addition, trismus and malocclusion hinder its widescale adoption. Anterior approaches, which include the transantral route, midface degloving, and lateral rhinotomy, create only a deep and narrow surgical field in the sinonasal cavity. Over the last two decades, great advances in nasoendoscopy have revolutionized patient care, and the application of the nasoendoscope has been promoted in the management of skull base tumors (10). Despite improving visualization, eliminating facial incision, and avoiding osteotomy, nasoendoscopic techniques, which are considered demanding procedures with steep learning curves, are usually associated with piecemeal resection. This compromises margin control and poses difficulties in managing intraoperative hemorrhage. Additionally, postoperative nasal morbidities such as nasal crusting, nasal obstruction, rhinorrhea, and an impaired sense of smell are not life-threatening but are objectionable and require long-term nasal care. Based on our review of the literature, only selected tumors with favorable histologies can be excised endoscopically (10–14).

Wei et al. first reported the maxillary-swing technique as an approach to persistent and recurrent tumors in and around the nasopharynx (1, 6). Otremba et al. have highly recommended adopting it to treat extensive ITF tumors, as it provides a broad view and poses minimal morbidity (3). However, most extensive tumors in this area protrude into the neighboring compartments at clinical onset. In addition, classic osteotomy protocol have the potential to corrupt the integrity of tumors, increasing the risk of tumor rupture, seeding, and consequent relapse. To overcome the pitfalls of the present method, we modified conventional osteotomies to achieve *en bloc* removal of such malignancies (Figure 1). First, the modified posterior osteotomy is initiated at the facial ridge and continued medially to the HP between the second premolar and the first molar. This bone cut is away from the primary site to avoid the risk of tumor rupture and protect the greater palatine artery during bone cutting in order to maintain a bloodless surgical field. Second, the anterior OF and infraorbital rim is rotated laterally, leaving *in situ* the posterior OF and infraorbital fissure, which are typically involved in tumors arising in the PPF due to their close topographical proximity.

After the partial maxilla is swung out, the remaining maxilla, the architecture of the sinonasal cavity and the OF can be seen under direct vision. According to Iannetti’s and Friedman’s theory (4, 15), the above-mentioned structures and the PP constitute the surgical planes of tumors in the PPF. The use of these boundaries can help surgeons define the physiological-cleavage planes to perform a truly oncological resection with adequate margins. However, we recommend removing them during surgical manipulation due to their inherent canals and foramina (e.g., sphenopalatine foramen, infraorbital fissure, and greater palatine foramen and canal), which probably serve as sanctuary sites for tumor cells (5). During tumor resection, brisk hemorrhage from the pterygoid plexus and internal maxillary artery is immediately encountered, and the surgical field is obscured by blood. In this circumstance, surgeons should take considerable caution to protect the internal carotid artery and eustachian tube from iatrogenic injury. These vital structures and the nearby condylar process are laterally located at the bottom of the surgical cavity created by the anterior approach. It should be noted that the maxillary-swing approach fails to resect the lateral part of the ITF, which is referred to as the “blind spot.” It represents a three-dimensional area circled by the coracoid process, condyle, and internal carotid artery (Figure 3D) (7). If malignancies involve or are close to this region, the ITF approach or a combined method is documented as an alternative surgical technique in these cases (7). After tumor removal, bleeding can be easily controlled by pressure packing or suture ligation due to the wide exposure.

The 14% (1/7) rate of locoregional recurrence we encountered in our study is within average ranges, compared with the outcomes of other techniques described in the literature (4, 13, 14, 16, 17). The MMS procedure exhibits some competitive advantages over those other approaches: (1) improved visualization of the PPF, sinonasocavity and skull base, which boosts surgical safety, helps stop bleeding and facilitates subsequent reconstruction by a free flap; (2) highlighting the principles of *en bloc* resection and removal of inherent canals and foramina around the tumor, which potentially reduce local recurrence; (3) preservation of the facial-nerve function and facial contours; and (4) minimizing postoperative trismus as the pterygoid muscle is resected. This modified procedure, however, has at least three drawbacks. First, the involvement of the “blind spot” impedes applications. Second, like the conventional way, the MMS causes cosmetic problems because of the incision in the upper lip. Third, there is a learning curve for undertaking this modified procedure.

Currently, summarizing the indications of the MMS approach would be premature due to the limited number of cases. Based on the analysis of the tumor characteristics we report in this study, there is a close correspondence between such abnormalities and the surrounding bony walls of the PPF, and all lumps extended to the ITF. Three of them protruded into the orbit via the infraorbital fissure, one extended to the oral cavity (OC) via the greater palatine foramen, two involved the MS, and three had eroded the lateral wall of the nose. This technique is therefore not only suitable for malignancies limited to the PPF but also for en bloc resection in cases of lesions that erupt into the ITF, MS, nasal cavity, orbit, or oral cavity, based on our preliminary practice. Under such circumstances, a rigorous preoperative evaluation of the disease with imaging studies should be conducted, and a multidisciplinary oncological institutional board should be assembled to seek consensus on the preferred treatment and surgical route, providing patients with the maximum benefit of expertise.

## Conclusion

In summary, the MMS approach is noteworthy in that it provides access to the PPF. Based on our practice, this approach offers good exposure to this deep region, permitting monobloc resection of extensive malignancies therein involving the ITF, MS, nasal cavity, orbit, or OC, with acceptable surgical morbidities and oncological outcomes. Future studies are needed to validate the reproducibility and efficiency of the MMS technique across larger case series and longer follow-up periods.

## Data Availability Statement

The raw data supporting the conclusions of this article will be made available by the authors, without undue reservation, to any qualified researcher.

## Ethics Statement

Written informed consent was obtained from the individual(s), and minor(s)’ legal guardian/next of kin, for the publication of any potentially identifiable images or data included in this article. The studies involving human participants were reviewed and approved by the Independent Ethics Committee of Hunan Cancer Hospital. Written informed consent to participate in this study was provided by the participants or their legal guardian/next of kin.

## Author Contributions

LX, WH, and JW contributed to the conception and design of the study. JW, YZ, JC, and XC contributed to the acquisition and analysis of the data. LX drafted the manuscript. All authors critically revised the manuscript, approved the final manuscript, and agreed to be accountable for all aspects of the works.

## Conflict of Interest

The authors declare that the research was conducted in the absence of any commercial or financial relationships that could be construed as a potential conflict of interest.
