# Supplementary material for: Modified Maxillary-Swing Approach for Resection of Primary Malignancies in the Pterygopalatine Fossa
Source: Front Oncol. 2020 Nov 9;10:530381. doi: 10.3389/fonc.2020.530381 (PMC7682189; doi:10.3389/fonc.2020.530381)
Supplement: Supplementary file 1 [file Data_Sheet_1.PDF]

Supplementary data

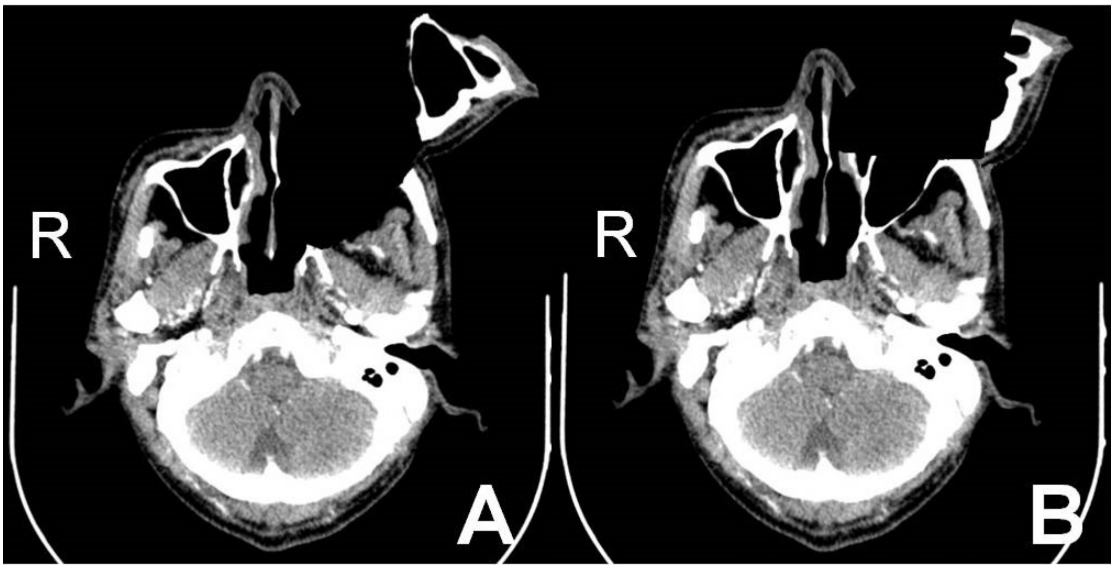

Supplementary figure 1 The schematic diagram (computed tomography) of the conventional maxillary swing approach (A) and the modified maxillary swing approach (B).

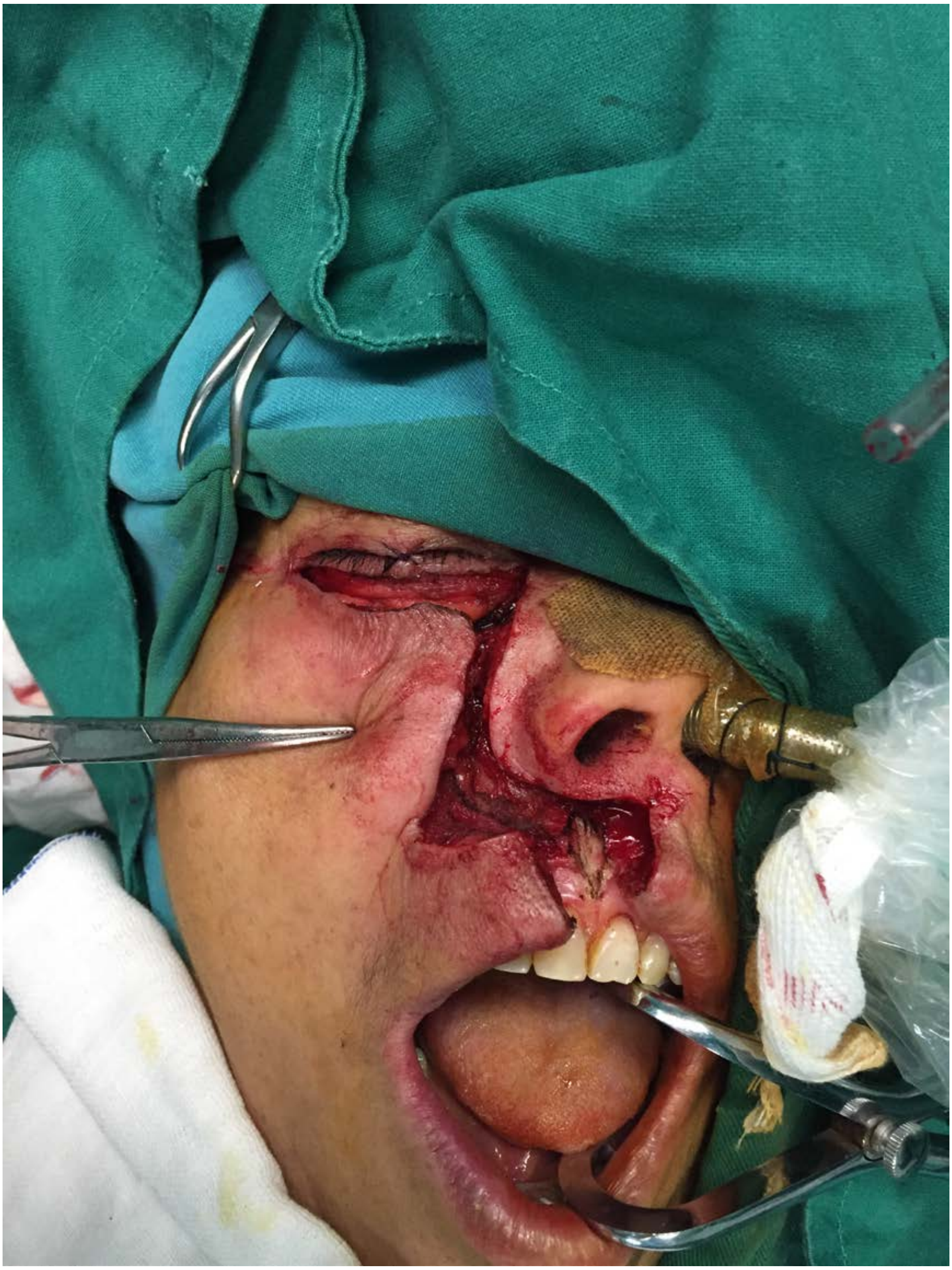

Supplementary figure 2A The Weber-Ferguson incision of the modified maxillary swing approach

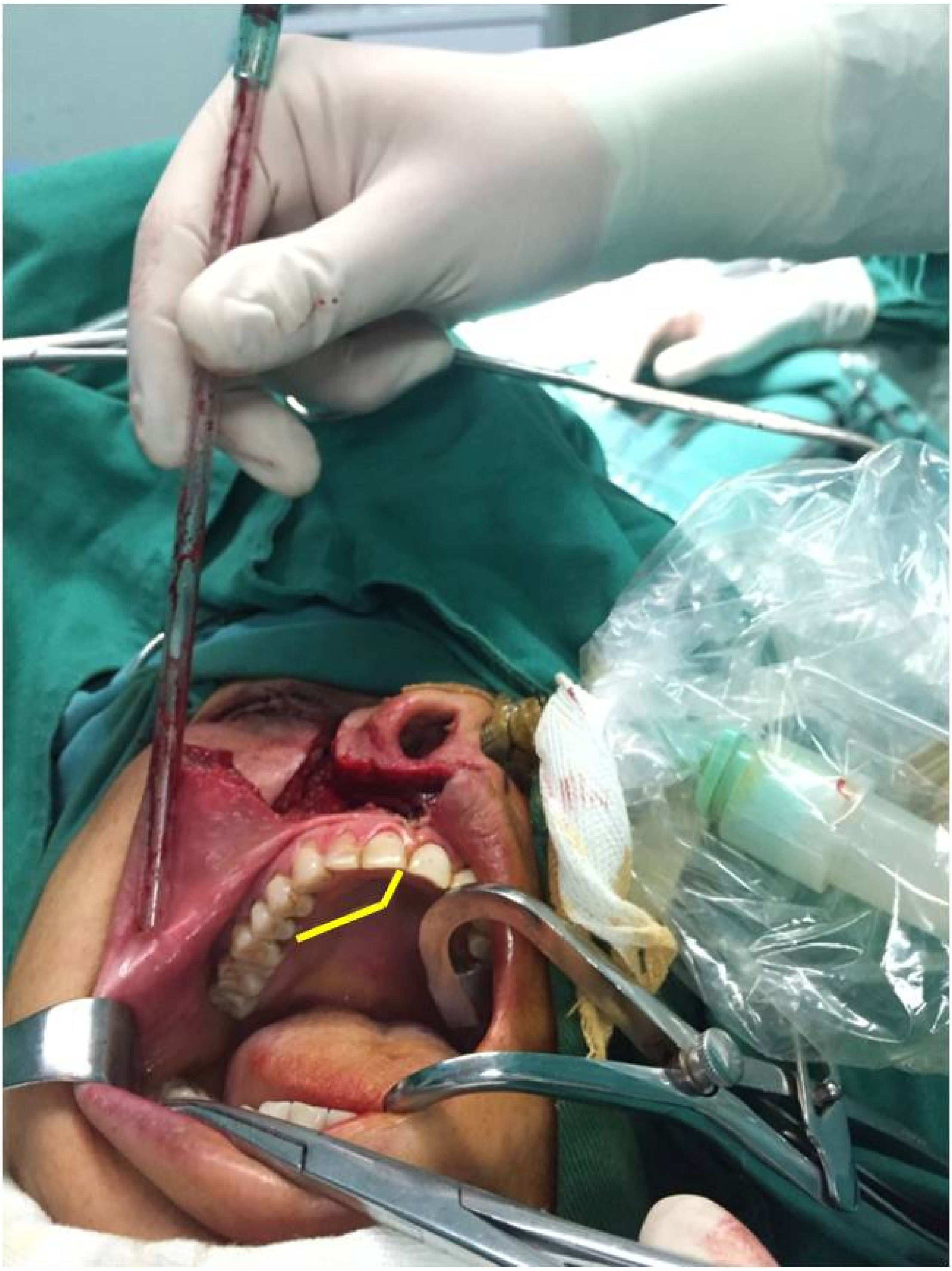

Supplementary figure 2B The mucosal incision of the hard palate is made in the midline and turned laterally to the gums between the second premolar and the first molar.

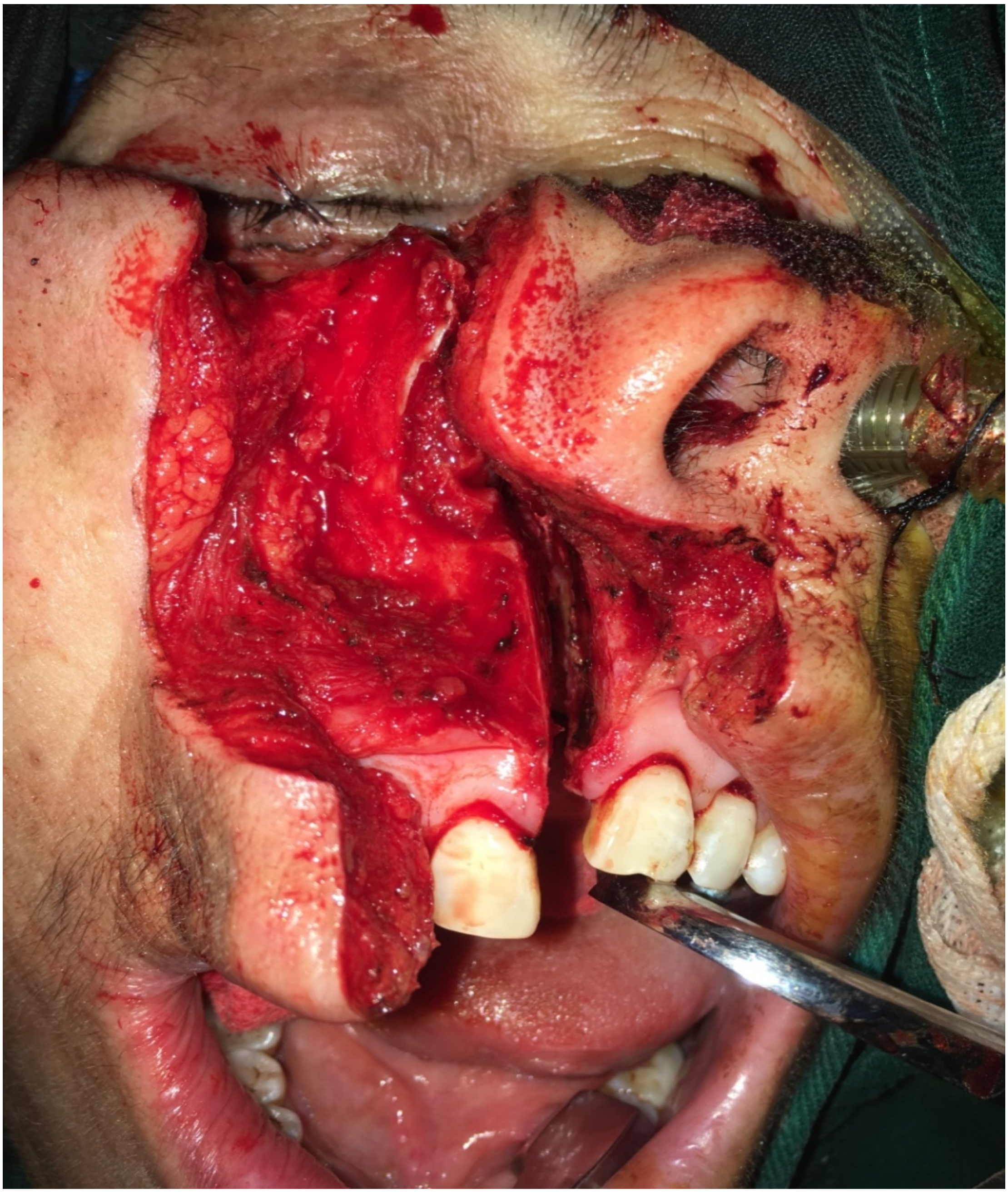

Supplementary figure 2C The anterior part of the maxilla is fractured.

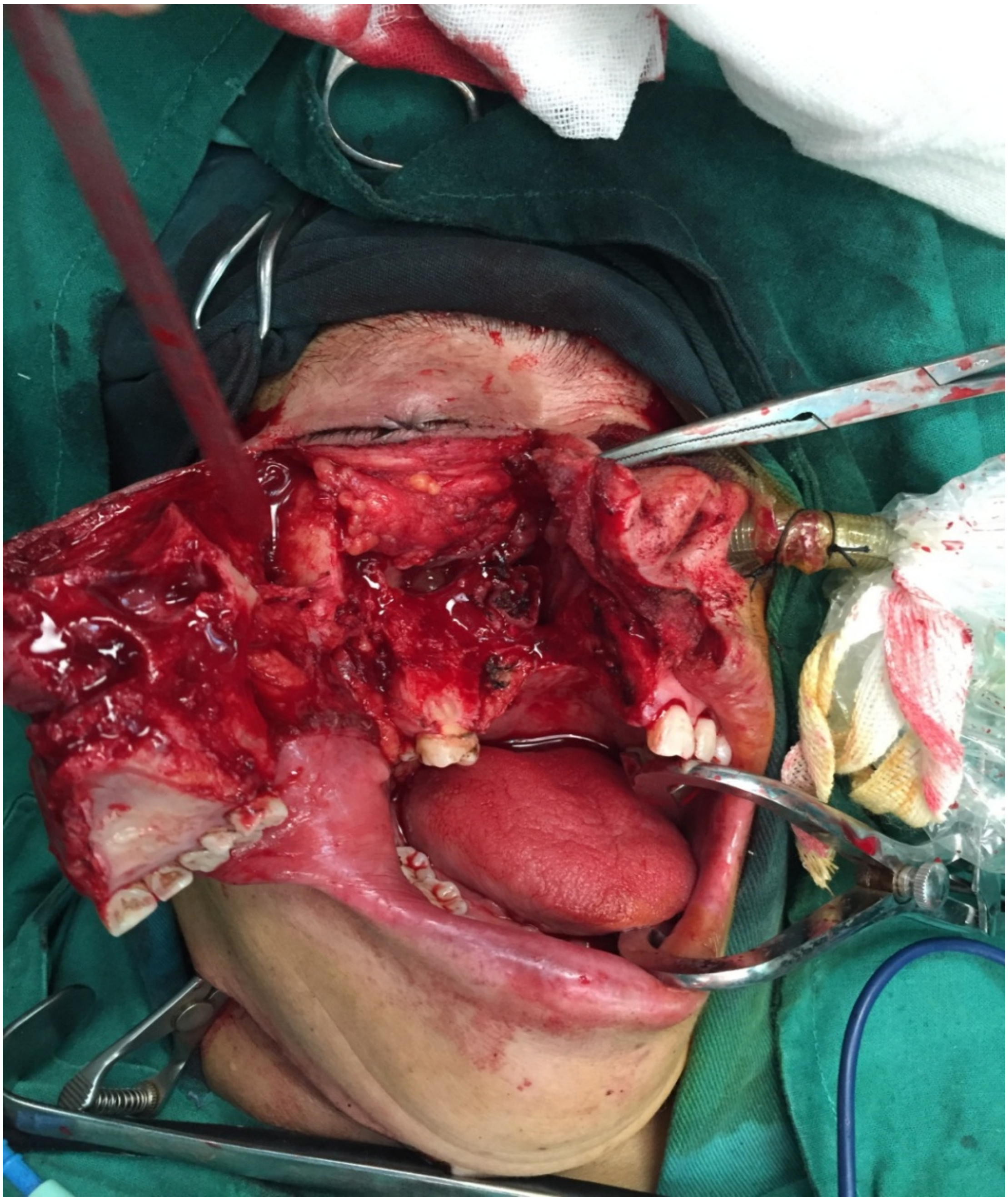

Supplementary figure 2D As the part of maxilla, orbital floor and infraorbital rim with facial skin are swung simultaneously, which results in a broad view of the remaining maxilla and the primary lesion.

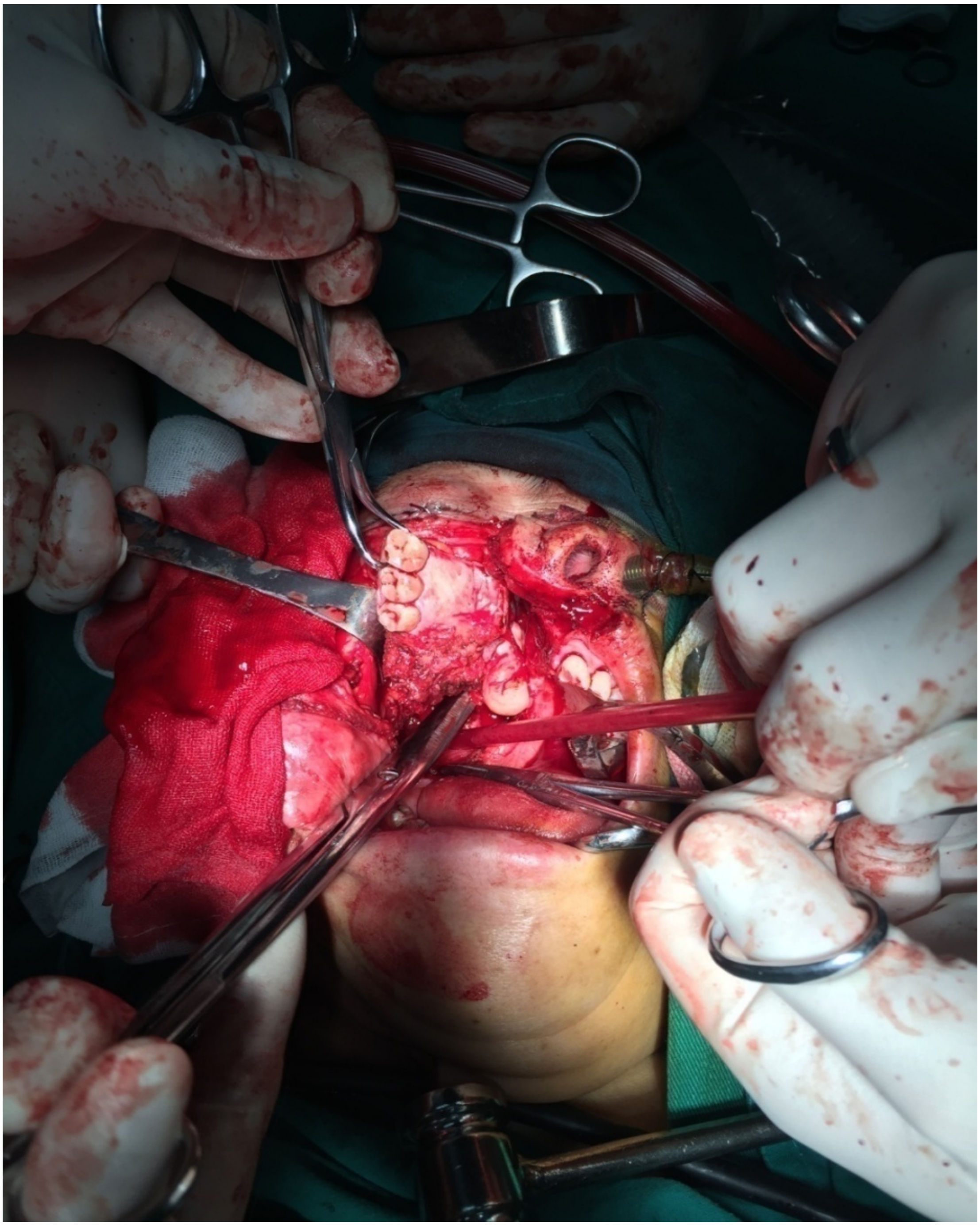

Supplementary figure 2E With the partial maxilla swung laterally, there is adequate space to allow scissors and other instruments employed to carry out truly en bloc resection of the primary malignancy in the pterygopalatine fossa.

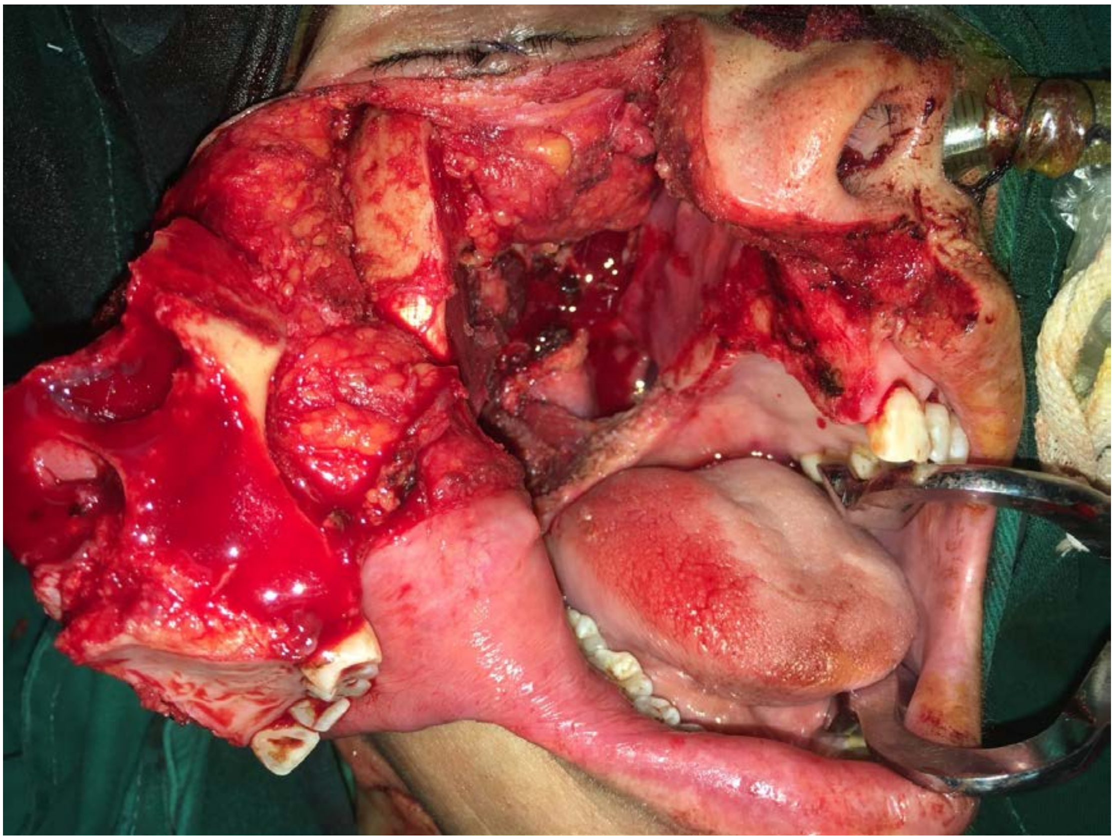

Supplementary figure 2F The panorama of the surgical field after removal of the tumor.

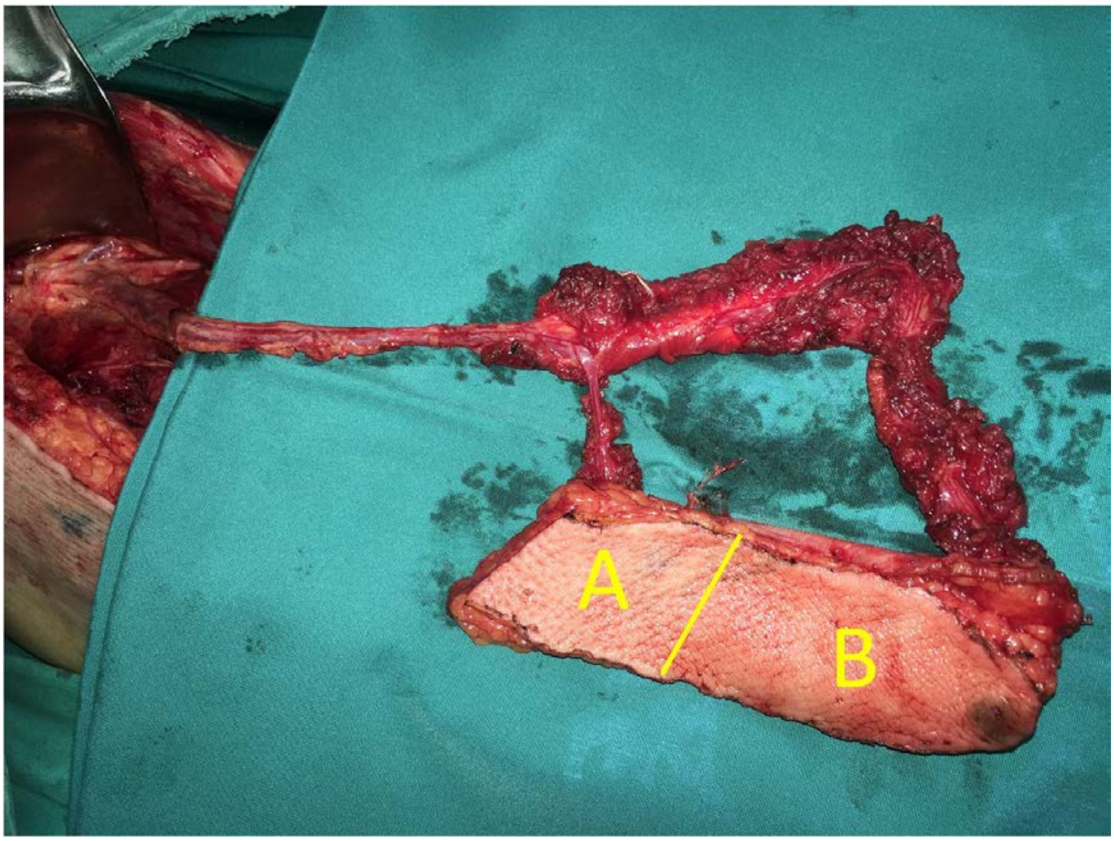

Supplementary figure 2G The anterolateral thigh flap is employed to reconstruct the defects (the lateral nasal wall, partial orbital floor and hard palate)

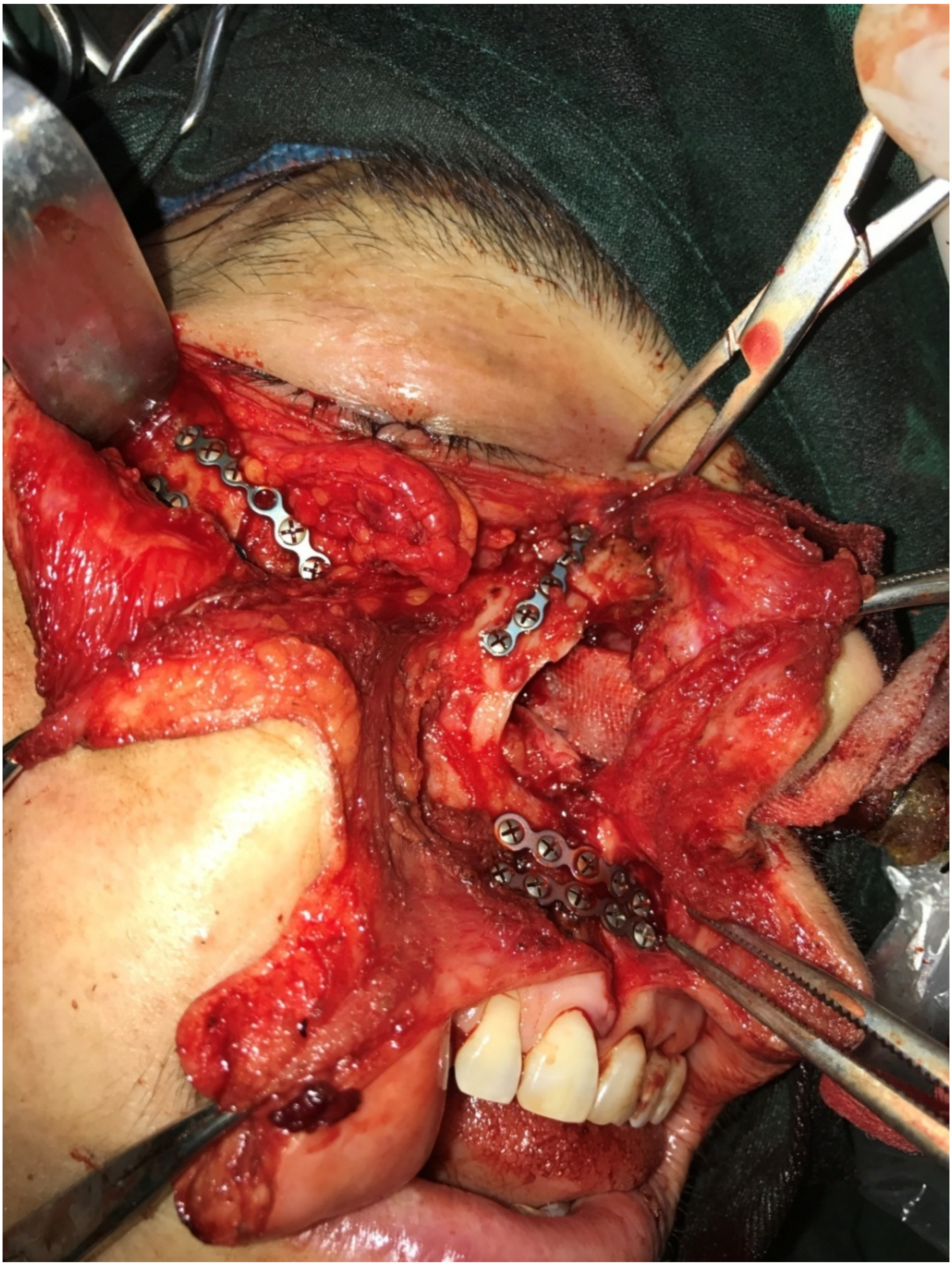

Supplementary figure 2H The laterally swung maxilla is then rotated back to its normal anatomic position and fixed to zygoma, frontal and alveolar process respectively with use of miniplates and screws.

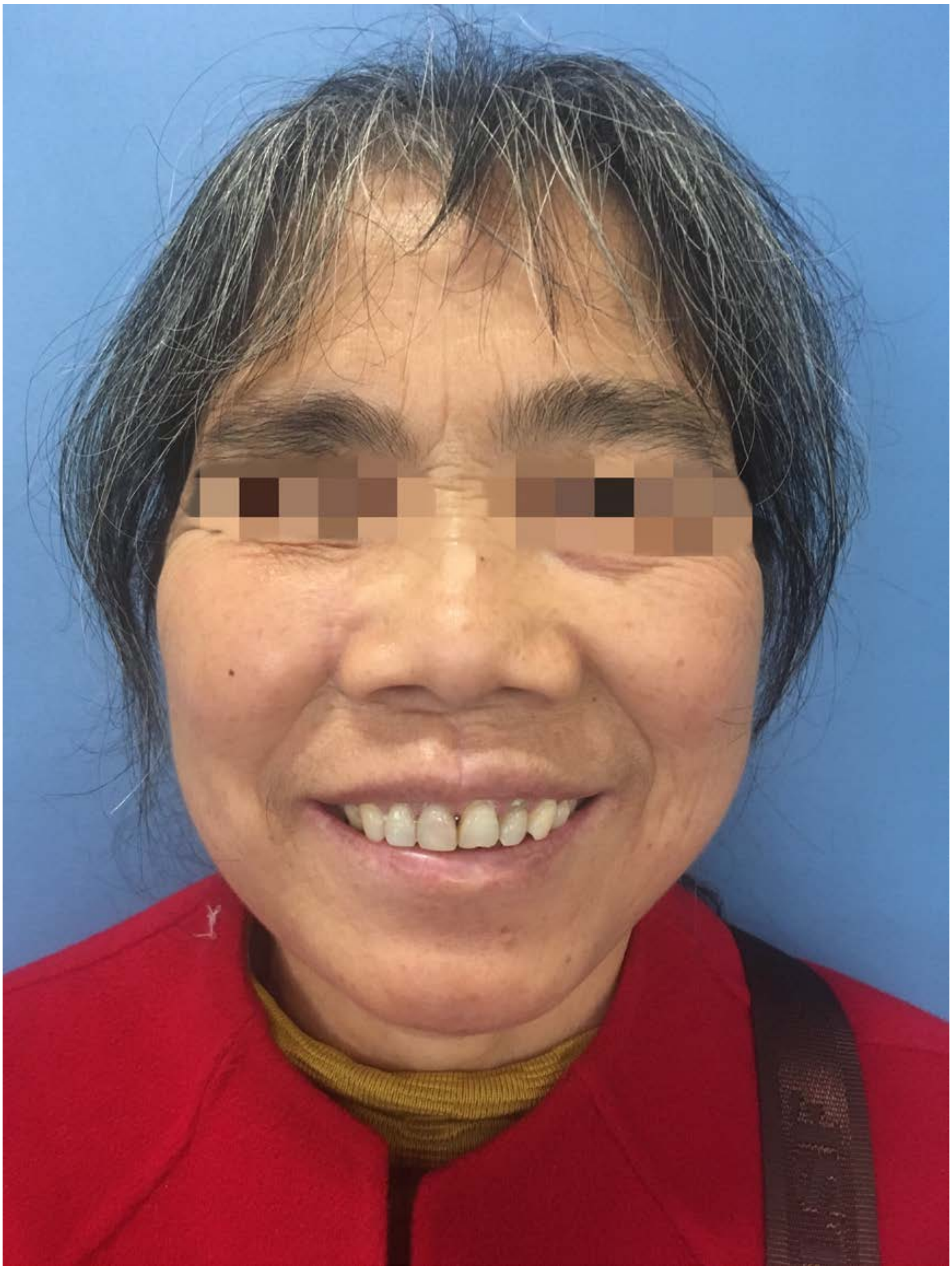

Supplementary figure 3 The facial scar is nearly invisible 12 months after surgery without any signs of facial palsy.
